# On Radical Cure of Hernia

**Published:** 1873-12

**Authors:** 


On the Radical Cure of Hernia.—Mr. Bryant, Surgeon to Guy's Hospital, believes that where a hernia can be kept up by a truss, and the patient is likely to remain in a civilized country where trusses can be obtained, any operation for the radical cure is unjustifiable. To risk the life of the patient on the theory of a cure, with the probability that he will be rendered less liable to its descent, when a truss has to be worn subsequent to the operation as a matter of safety, is, he says, a practical delusion.—*The Practice of Surgery: a Manual. London.* 1872.

